# Beyond the visible spectrum – applying 3D multispectral full-body imaging to the VirtoScan system

**DOI:** 10.1007/s12024-021-00420-x

**Published:** 2021-09-17

**Authors:** Sören Kottner, Martin M. Schulz, Florian Berger, Michael Thali, Dominic Gascho

**Affiliations:** 1 Zurich Institute of Forensic Medicine, University of Zurich, Winterthurerstrasse 190/52, 8057 Zurich, Switzerland; 2 Institute of Forensic Medicine, Ludwig-Maximilians-University Munich, Nussbaumstrasse 26, 80336 Munich, Germany

**Keywords:** Photogrammetry, Infrared photography, Ultraviolet photography, Forensic imaging, Photographic documentation, Multicamera setup

## Abstract

Multispectral photography offers a wide range of applications for forensic investigations. It is commonly used to detect latent evidence and to enhance the visibility of findings. Additionally, three-dimensional (3D) full-body documentation has become much easier and more affordable in recent years. However, the benefits of performing 3D imaging beyond the visible (VIS) spectrum are not well known, and the technique has not been widely used in forensic medical investigations. A multicamera setup was used to employ multispectral photogrammetry between 365 and 960 nm in postmortem investigations. The multicamera setup included four modified digital cameras, ultraviolet (UV) and near-infrared (NIR) light sources and supplemental lens filters. Full-body documentation was performed in conjunction with the use of a medical X-ray computed tomography (CT) scanner to automate the imaging procedure. Textured 3D models based on multispectral datasets from four example cases were reconstructed successfully. The level of detail and overall quality of the 3D reconstructions varied depending on the spectral range of the image data. Generally, the NIR datasets showed enhanced visibility of vein patterns and specific injuries, whereas the UV-induced datasets highlighted foreign substances on the skin. Three-dimensional multispectral full-body imaging enables the detection of latent evidence that is invisible to the naked eye and allows visualization, documentation and analysis of evidence beyond the VIS spectrum.

## Introduction

In postmortem forensic examinations, digital photography is most commonly utilized to capture the visible (VIS) part of the electromagnetic spectrum. As a result, the findings are documented in the same way they were perceived by the human eye at the time of the examination. Therefore, it is not surprising that textures used for three-dimensional (3D) surface documentation are usually also captured within the VIS spectrum [1–24]. However, the literature shows a variety of possible applications of forensic photography on either side of the VIS part of the electromagnetic spectrum and with the help of narrowband light sources.

The VIS part of the electromagnetic spectrum (i.e. visible light or visible radiation) lies between a lower limit of approximately 360 and 400 nm (nm) and an upper limit of approximately 760 and 830 nm [25]. Ultraviolet (UV) radiation is characterized by wavelengths shorter than those of VIS light, ranging from 100 to 400 nm [25]. In contrast, near-infrared (NIR) radiation (also referred to as IR-A) is characterized by wavelengths longer than those of VIS light. NIR radiation ranges from approximately 780 nm to 1400 nm [25]. When electromagnetic radiation within the range from UV to NIR hits matter, it is reflected or refracted at the surface. The refracted electromagnetic radiation is scattered and re-emitted or absorbed. When UV radiation is absorbed by matter and causes excitation of a molecule, the stored energy is re-emitted as electromagnetic radiation with a longer wavelength as soon as the molecule relaxes. The difference between the band maxima of the absorption and emission spectra is known as the Stokes shift [26–28]. When only emission occurs, as long as the UV-emitting light source is switched on, the emission is referred to as fluorescence. This behavior is used for UV-induced fluorescence photography.

Within the field of forensic medicine and pathology, one of the applications most commonly associated with multispectral photography is the detection and visualization of latent bruises and injuries [29–43]. Some articles have reported the usefulness of multispectral photography for the detection and documentation of occult bruises in children [30, 35, 37, 42, 43] and adults [32, 35, 37, 41]. In addition, some studies have specifically referred to the use of multispectral photography to visualize and analyze bite marks [31, 33] and bruises in connection with strangulation [34]. In addition to its application in the investigation of bite marks, multispectral photography has been used in various areas in forensic odontology. For example, multispectral photography has been used to detect and visualize dental restorations [44–46] and to investigate the age-dependent properties of dental fluorescence [47]. Another topic linked to multispectral photography is the detection and visualization of bodily fluids. Some studies have investigated the potential of different wavelengths to allow bodily fluids to be distinguished from other substances, such as liquids, gels and ointments [48–50]. Furthermore, studies and case reports have described the usefulness of different wavelengths to visualize latent bodily fluids on textiles [48, 49, 51] and human skin [48, 50, 52–54]. UV fluorescence has been applied to detect cryptic skin particles on various materials [55]. NIR photography has the potential to detect and visualize tattoo ink on human skin after advanced decomposition [31, 41, 56–61]. This application has been shown to assist in the successful identification of the deceased [58, 59, 61]. Furthermore, multispectral photography has been used to investigate tattoo modifications and to determine whether tattoos were used to cover older tattoo designs [62, 63]. Additionally, NIR imaging has been shown to improve the visualization of vein patterns in the human body [64–69]. For example, images showing palm vein and palm dorsum vein patterns have been used for biometric verification and human identification [65, 66, 69, 70].

In summary, the main goal of multispectral imaging in a forensic context is to detect, enhance, visualize and document latent evidence that is barely perceptible or invisible to the human eye. The greatest challenge for multispectral imaging is locating the evidence, since it can easily be overlooked. The search can be time consuming, especially when it involves large and complicated surfaces such as those of an entire body. Moreover, the need to use a range of spectra can prolong the procedure. Since time is a critical factor in any forensic investigation, the use of fast 3D multispectral imaging could be of help, as it would require the use of the body only for the duration of multispectral 3D documentation. Immediately thereafter, the body would be available for other examinations while the search for latent evidence is conducted digitally. A digital 3D model allows interaction with and navigation through the data, which can facilitate locating latent evidence and contribute to a better understanding of the dataset and its spatial relationships [71]. In addition, 3D data can be used to acquire measurements on the body [14, 18, 19, 22]. Finally, 3D data can be used for 3D reconstructions of crime and accident scenes [6, 7, 9, 24] to perform matching between an injury and an injury-causing object [1–5, 71].

However, it seems that the benefits of 3D multispectral imaging are not yet well known, as this technique has not been widely used in forensic investigations. To the best of our knowledge, there is only one publication to date reporting the potential use of 3D multispectral photogrammetry for forensic investigations at the crime scene. In that publication, Edelman and Aalders [72] describe a method for close-range, single-camera, thermal, multispectral and hyperspectral photogrammetry for crime scene documentation. Studies regarding the use of 3D multispectral imaging for forensic-medical full-body examinations seem to be absent in the scientific literature.

To address this absence, we aimed to develop an automated, fast and cost-effective multispectral 3D imaging system for postmortem examinations. The objective of this study was to investigate the feasibility of performing semiautomatic 3D multispectral full-body documentation using photogrammetry. Furthermore, we aimed to investigate whether 3D surface documentation beyond the VIS spectrum could provide information beyond that acquired by standard postmortem 3D surface documentation within the VIS spectrum. We used NIR illumination for imaging solely in the NIR range and UV illumination for imaging in the VIS to NIR range (referred to as UV-induced UV-I imaging in this study).

## Methods and materials

### Hardware setup

**Fig. 1:**
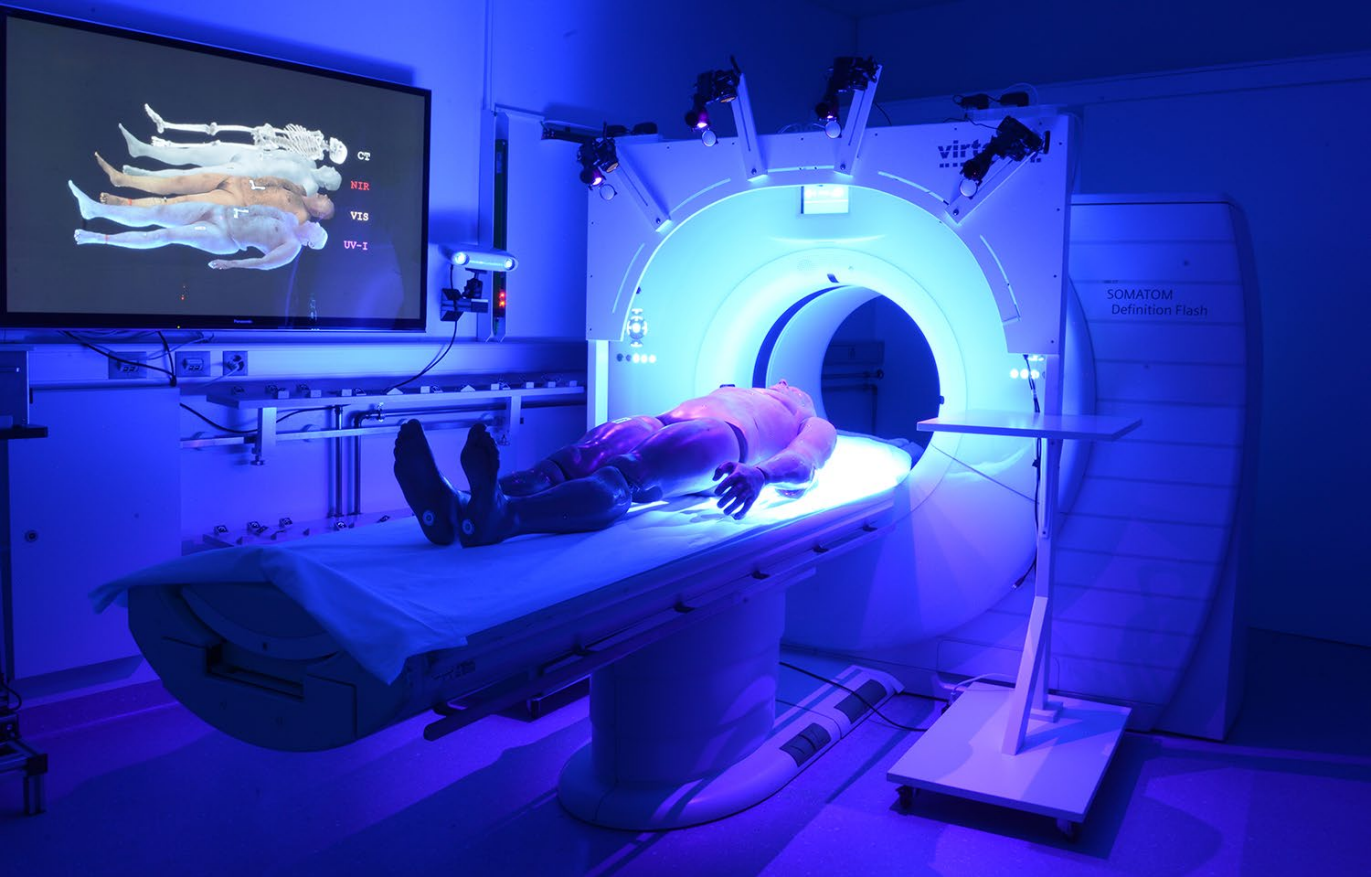
Photo of the multispectral imaging setup in front of the CT gantry. In this photo, the multicamera rig is equipped with UV light sources and yellow lens filters with 50% transmission at 460 nm

A multicamera setup was used to perform multispectral full-body photogrammetry. The construction and design of the multicamera setup are described in our previous reports [13, 15]. The camera rig was based on a wooden mobile frame. Four digital single lens reflex (DSLR) cameras (EOS 200D, Canon Inc., Tokyo, Japan) were used in combination with a Canon EF lens with a 40 mm fixed focal length (EF 40 mm f/2.8 STM, Canon Inc.). All camera filters in front of the sensor were removed and replaced by a multispectral glass filter (transmission range: 190–4000 nm) in advance by a camera specialist (Optic Makario GmbH, Mönchengladbach, Germany). The multispectral glass filter was added to access the entire spectral capacity of the camera sensor, thereby preventing dust contamination on the sensor and restoring focus functionality. Additionally, each camera was equipped with a wireless remote shutter control (Yongnou RF-603C II, YongNuo Photographic Equipment Co., Ltd., Shenzhen, China). All cameras were mounted on tripod ball heads (BALL 19P, NOVOFLEX Präzisionstechnik GmbH, Memmingen, Germany), which were attached to slider boards on the mobile frame. The combination of slider boards and ball heads allowed the cameras to be positioned in an arch-like manner and ensured that overlapping images were created along the entire width of the body. For the VIS, UV-I and NIR image series, each camera was equipped with a specific set of lens filters and, in the case of the UV-I and NIR setup, additional light sources (Tables 1 and 2). An image of the multicamera setup equipped with the UV light sources is provided in Fig. 1.

**Table 1:** Tunable UV and NIR light sources used for multispectral photogrammetry

Manufacturer	Product name	Wavelength [nm]	Class
Dedolight	DLOBML-BI-UV Fluoreszilla	365—400	UV
Dedolight	DLOBML-BI-IR iREDZILLA	860—960	NIR

**Table 2:** Lens filters used for multispectral photogrammetry. Detailed information about the IR-neutralization f ilters is not available. According to the manufacturer, IR-neutralization filters restore the original camera transmission range and color representation

Manufacturer	Filter name	50% transmission [nm]	Approx. transmission range [nm]
Optic Makario	yellow	460	450–1100
Optic Makario	IR neutralization	N/A	Original camera transmission range
Optic Makario	IR longpass	850	810–1100

### Image-capturing process

Multispectral photogrammetry was carried out in a semiautomatic manner in conjunction with the use of a medical X-ray computed tomography (CT) scanner (Somatom Definition Flash, Siemens Healthineers, Erlangen, Germany). First, the multicamera rig was placed in front of the CT gantry and aligned centrally above the CT couch. Subsequently, during the scanning procedure, the operating software of the CT scanner (Syngo CT 2012B, VA44A, Siemens Healthineers, Erlangen, Germany) was used to move the CT couch underneath the multicamera rig. To acquire overlapping images along the entire length of the body, the CT couch was moved in increments of 10 cm. Between moves, all cameras were triggered simultaneously with the remote shutter release. This procedure was executed for each of the VIS, UV-I and NIR image series separately.

The multicamera setup had to be adapted for each image series. In the case of the VIS image series, an absorptive filter called an IR-neutralization filter was attached to each camera lens. This filter restores the default transmission characteristics from the original camera configuration. During the VIS image series, standard ceiling lighting was used as the light source. For the UV-I image series, a yellow filter with 50% transmission at 460 nm was attached to each camera lens (Table 2). In addition, a tunable UV light source (DLOBML-BI-UV Fluoreszilla, Dedolight Forensic Series, Dedo Weigert Film GmbH, Munich, Germany) was attached to each camera. For the NIR image series, a longpass filter with 50% transmission at 850 nm was applied to each camera lens. In addition, a tunable NIR light source (DLOBML-BI-IR iREDZILLA, Dedolight Forensic Series, Dedo Weigert Film GmbH, Munich, Germany) was attached to each camera. The tunable UV and NIR light sources offered a range of wavelengths (UV: 365–400 nm, NIR: 860–960 nm). In both cases, image series were conducted with the minimum and maximum values (Table 1). For the UV-I and NIR image series, any additional light source was turned off during the image-capturing procedure.

The camera settings for ISO (ISO 100) and aperture (f/16) were maintained constant throughout the study (Table 3). However, the exposure times, white balance and camera focus had to be adjusted manually for each image series.

**Table 3:** Camera settings for the VIS, UV-I and NIR image series

Parameter	Setting
Iso value	100
Aperture	f/16
White balance	Custom WB
File type	JPG
Camera focus	Manual mode

### Multiview 3D reconstruction and 3D model optimization

Subsequent to image acquisition, all photos were transferred to a mobile workstation (Lenovo ThinkPad P53, Lenovo Group Limited, Hong Kong, China; 12 Intel(R) Core(TM) i7-9850H CPUs with 6 cores at 2.60 GHz, Intel Corporation, Santa Clara, USA; 128 GB RAM, NVIDIA Quadro RTX 5000 GPU, NVIDIA Corporation, Santa Clara, USA; Microsoft Windows 10 operating system, Microsoft Corporation, Redmond, USA). Agisoft Metashape Professional (Version 1.6.1 build 10,009, Agisoft LLC, St. Petersburg) was used for the multiview 3D reconstruction of the captured photogrammetric images. The 3D reconstructions were computed based on default settings for high-resolution models. Textures were calculated based on a size of 12,288 pixels. In some of the image series, the initial alignment of the photos failed. In those cases, the image set of that particular image series was divided in half. Subsequently, both parts (chunks) of the divided image set were reconstructed separately. Finally, both chunks were merged into one complete data set, and the standard reconstruction procedure was continued.

After the 3D reconstructions were finished, several point-to-point distances from reference scale bars were used to scale the 3D models. This procedure was performed with the help of Agisoft Metashape Professional. After that, all 3D models were edited using 3D inspection software (GOM inspection suite, Version 2020, Hotfix 1, Rev. 131,819, Build 2020–10-12, GOM GmbH, Braunschweig, Germany). During the editing process, the boundaries of the mesh were cleaned, and parts of the mesh that held no additional useful information, such as the surface area of the CT couch, were deleted. The editing process helped to reduce the size of the datasets. Finally, with the help of Agisoft, textures in the edited 3D models were calculated.

### Evaluation and selection of forensic cases

Overall, five forensic cases (male: n = 2; female: n = 3) were used to investigate the applicability of 3D multispectral full-body imaging. All cases were selected randomly. Bodies with clear signs of advanced decomposition were excluded from the study. The first case was used to adjust the camera settings and to prepare a protocol for the imaging procedure. This case was not included in the results of this study. 3D multispectral full-body imaging of the four subsequent cases was conducted according to the imaging protocol. All cases and results were visually analyzed and evaluated by an engineer with experience in 3D metrology, 3D imaging and analysis, and forensic photography. Findings were reviewed in consultation with a forensic pathologist. However, none of the findings presented in the results were confirmed by histology or any further analysis.

## Results

An imaging protocol for 3D multispectral full-body imaging was used to document four exemplary forensic cases. For each case, five photogrammetric datasets were recorded. Altogether, 20 photogrammetric datasets were included in this study. On average, image acquisition for the entire body took approximately 5 min for the UV-I image series and approximately 3 min for each of the VIS or NIR image series.

### Multiview 3D reconstruction

**Fig. 2:**
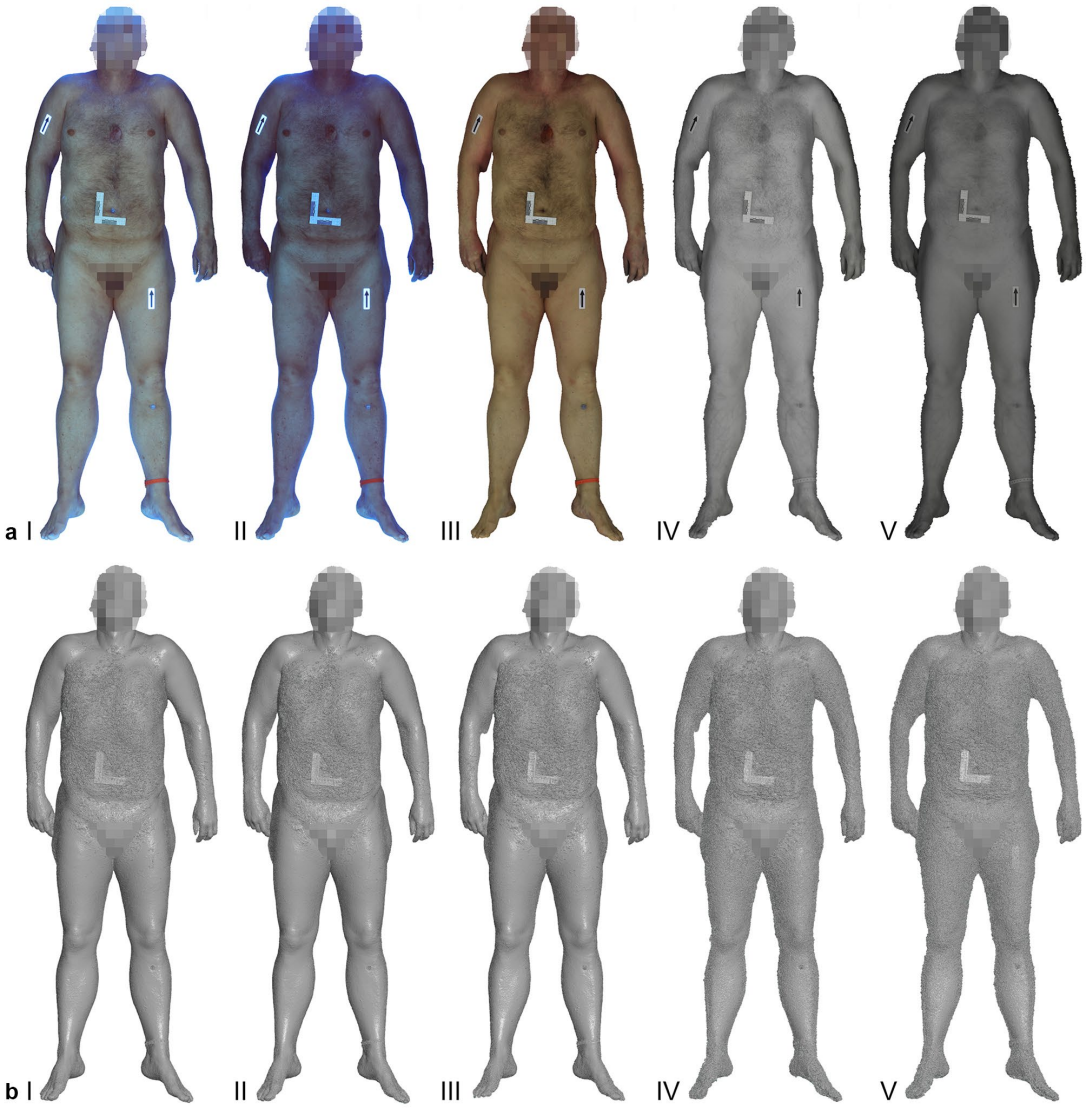
Comparison of the textured 3D models (**a** I-V) and the corresponding 3D polygonal mesh representations (**b** I-V) from the entire multispectral imaging set for case one. Images a I and b I refer to the UV-I dataset at 365 nm. Images a II and b II refer to the UV-I dataset at 400 nm. The VIS dataset is represented by images a III and b III. The remaining image sets of a IV/b IV and a V/b V refer to the NIR dataset at 860 nm and 960 nm, respectively

Textured 3D models were reconstructed successfully for all image series. An example of textured models and their corresponding 3D polygonal mesh representation is presented in Fig. 2. The 3D reconstructions for the VIS and UV-I image series produced a comparable level of detail regarding the 3D polygonal meshes (Fig. 2b I-III). The 3D reconstructions from the NIR image series exhibited a higher level of noise and contributed to a rougher representation of the 3D object (Fig. 2b IV-V). This pattern was noted in all cases.

### Comparison between the VIS and NIR datasets

**Fig. 3:**
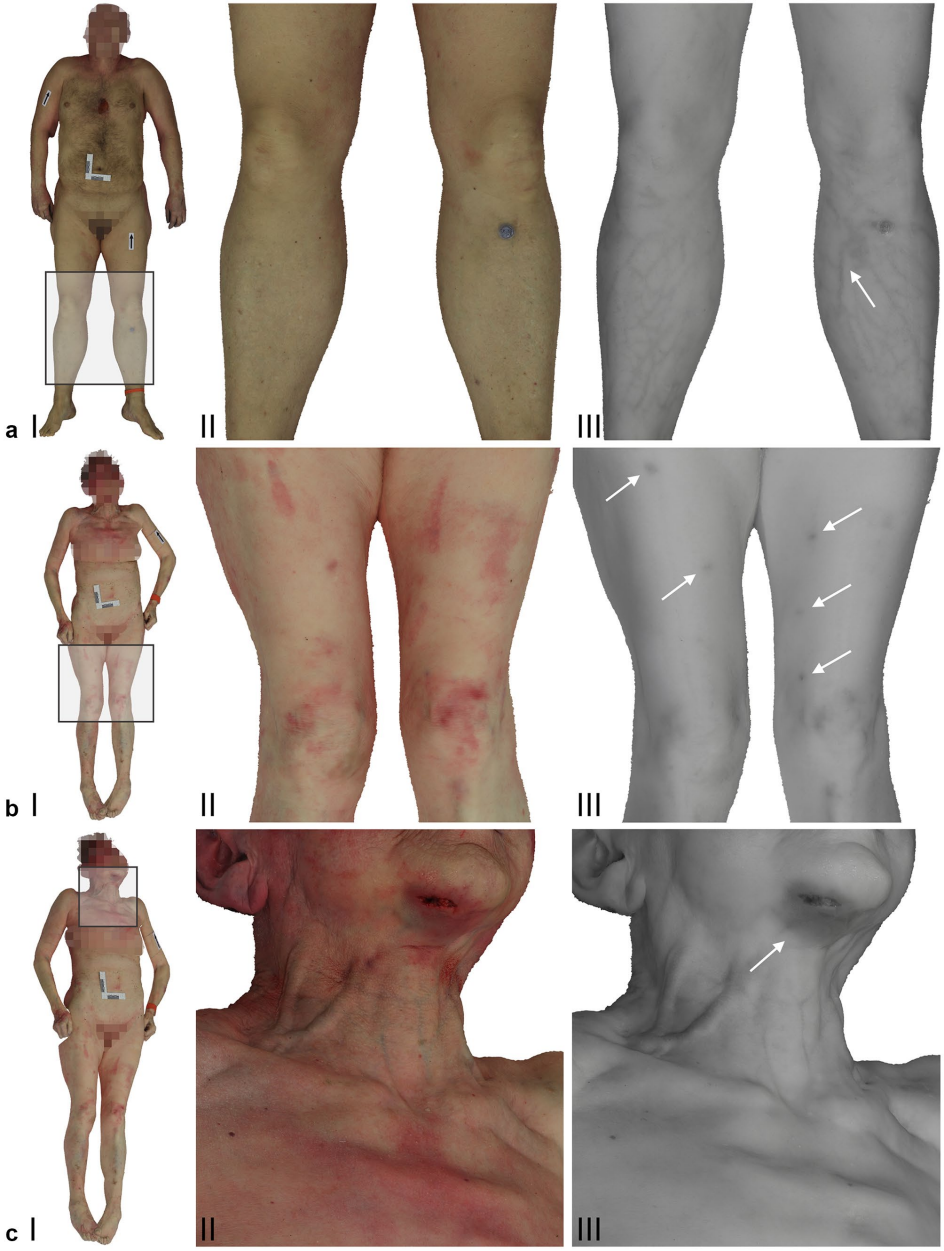
Screenshots from the 3D models based on the VIS setup (**a** I-II, **b** I-II, **c** I-II) and the NIR setup at 860 nm (**a** III, **b** III, **c** III). Images a II and a III illustrate the lower extremities from case one. The NIR image a III presents a vein pattern, which is indicated by the white arrow. Images b II and b III represent the lower extremities from case three. The arrows in the NIR image b III indicate hematomas. Images c II and c III show the chin, neck and upper torso from case three. The arrow in the NIR image c III indicates a hematoma on the chin

A comparison between the VIS and NIR textures of the 3D datasets showed that contrast, discolorations and certain characteristics regarding the skin and surface of the body were represented differently between the two spectra (Fig. 3). For instance, nevi were perceptible in the VIS datasets but were often absent in the corresponding NIR datasets. On the other hand, vein patterns in the extremities, such as the lower legs or the anterior of the lower arms, exhibited high contrast in the NIR datasets, whereas vein patterns in the corresponding VIS datasets had low contrast and weak visibility (Fig. 3a, b). Light red discolorations on the skin were detectable in the VIS datasets but were absent in the NIR datasets. However, darker, somewhat bluish discolorations evident in the VIS datasets also appeared with discernible contrast in the NIR datasets (Fig. 3c-f). A summary of these findings is provided in Table 4.

**Table 4:** Quality and differentiability of the visual representation of selected features according to the image series and spectra. Numbers from 0–3 refer to the quality and differentiability: 0 = not visible, 1 = vaguely visible, 2 = visible but hardly differentiable, and 3 = clearly visible and differentiable

Features	UV-I		VIS	NIR	
	**365 nm**	**400 nm**		**860 nm**	**960 nm**
Vein patterns	1	1	1	3	3
Bruises (light red)	2	2	2	0	0
Bruises (dark red, bluish)	2	2	2	3	3
Bodily fluids	1	3	0	0	0
Antiseptic cleansing agent	3	3	3	0	0
Adhesives	3	3	1	0	0

### Comparison between the VIS and UV-I datasets

**Fig. 4:**
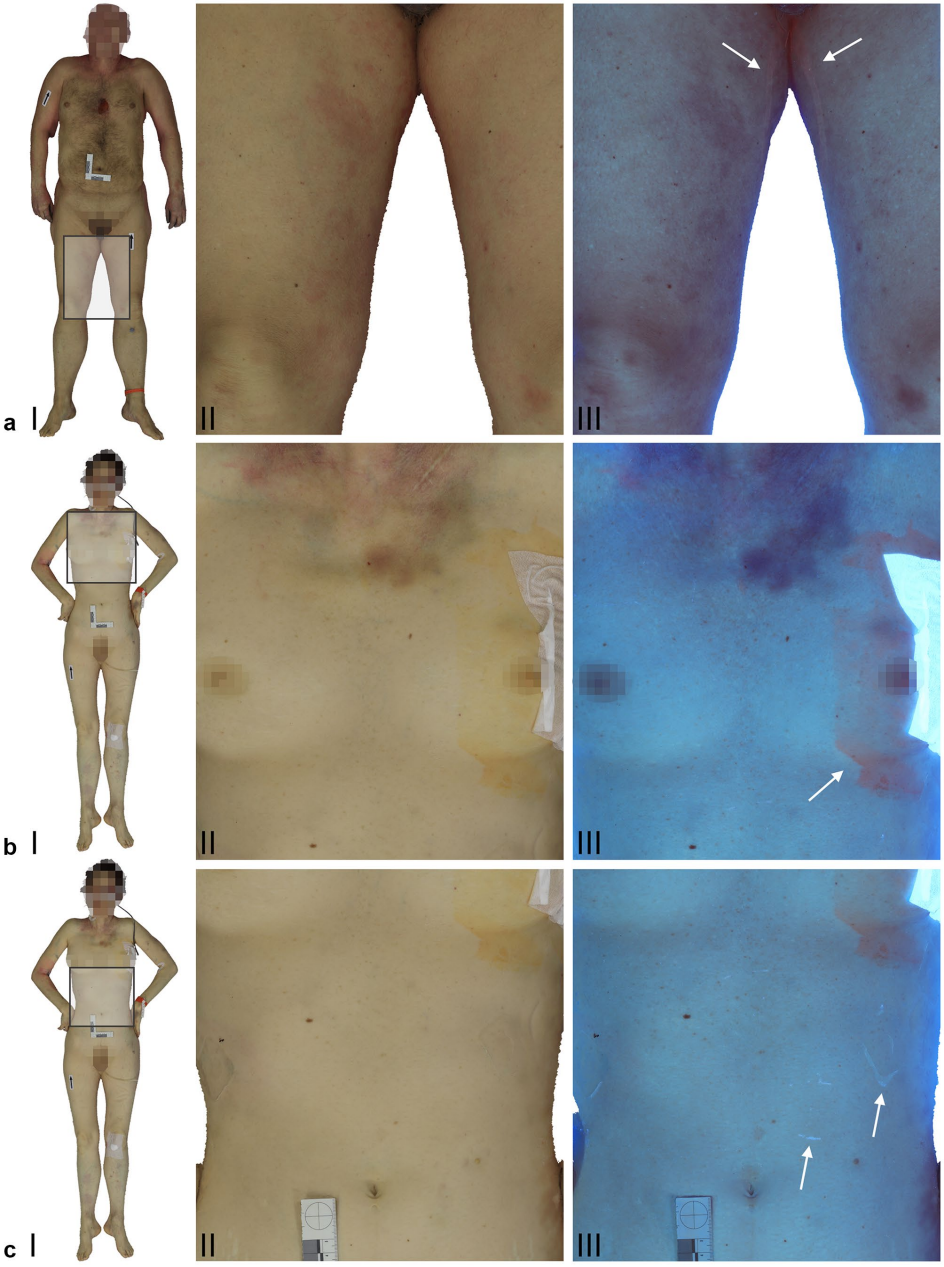
Screenshots from the 3D models based on the VIS setup (**a** I-II, **b** I-II, **c** I-II) and the UV-I setup at 400 nm (**a** III, **b** III) and 365 nm (**c** III) in combination with a yellow filter. Images a II and a III illustrate the inner thigh from case one. The arrows in the UV-I image a III indicate a trace of dried bodily fluid. Images b II and b III represent the upper torso from case two. The arrow in the UV-I image b III indicates an antiseptic cleansing agent. Images c II and c III display the abdomen from case two. The arrows in the UV-I image c III indicate residue from applied adhesives

A comparison between the VIS and UV-I textures of the 3D datasets showed that certain characteristics on the body surface and qualities of the skin were depicted differently between the two spectra (Fig. 4). For example, discolorations on the skin were discernable in the VIS datasets. However, in the UV-I datasets, the texture information on the body surface seemed more saturated and in most cases exhibited higher contrast than that of the VIS datasets regarding the discolorations. Hematomas, which were displayed as red and bluish discolorations in the VIS datasets, appeared darker and more distinct in the UV-I datasets. On the other hand, the color differentiation evident in the VIS datasets was not apparent in the UV-I datasets. Images a and b in Fig. 4 display a trace of a potentially dried bodily fluid. In the UV-I dataset, the trace was highlighted and visible, whereas in the corresponding VIS datasets, the trace was not initially evident. A detailed view of the VIS dataset displayed a slight difference in surface texture that indicated the trace. In images c and d in Fig. 4, an antiseptic cleansing agent was visible in both datasets. The saturation and contrast of the UV-I dataset appeared higher and more distinct than those of the VIS dataset. Images e and f in Fig. 4 illustrate residue from applied adhesives. In the VIS dataset, the location of the applied adhesives was predominantly visible due to indentations left on the skin. In the UV-I dataset, the residue from the adhesives was highlighted and exhibited high contrast, whereas the indentations were less apparent. An overview of these findings can be found in Table 4.

## Discussion

In this study, a method for fast 3D multispectral full-body imaging was developed. Photogrammetric datasets were acquired semi automatically in conjunction with the use of a medical CT scanner. Furthermore, a workflow to produce textured 3D models based on multispectral image data using a popular type of photogrammetry software was presented. The data quality of 3D reconstructions based on four example cases was analyzed. Additionally, features on the body surface that showed apparent disparities among the VIS, UV-I and NIR datasets were evaluated.

All of the acquired datasets were reconstructed successfully with the help of Agisoft Metashape Professional. The results showed that the 3D reconstructions varied in quality and level of detail depending on the spectral range of the image data. The 3D reconstructions of the UV-I and VIS datasets seemed to perform comparably well. Based on visual comparison, the 3D mesh representations exhibited a similar level of detail. In contrast, the 3D reconstructions of the NIR datasets generally showed a lower level of detail. The corresponding 3D models showed noisy 3D mesh representations featuring rough surfaces. This observation was most likely due to the characteristics of NIR radiation and its representation in the NIR images. The NIR images in this study mainly displayed body hair, vein patterns and injuries. In addition, the bodies in the NIR images had a largely homogenous texture. Such a texture is impractical for photogrammetric reconstructions that require non-uniform surfaces with sufficient texture information. Hence, 3D reconstructions based on NIR images exhibited noisy surfaces that contributed to a generally lower level of detail.

Generally, Agisoft Metashape Professional offers functionalities for datasets and image stacks based on multiple wavelengths [72]. However, the setup used in this study could not guarantee that the camera positions and angles remained unchanged throughout the imaging procedure. It is possible that positions and angles were modified slightly during the manual replacement of the filters and the light sources. For this reason, the 3D reconstructions were calculated separately for each dataset rather than mapping different textures onto one selected 3D reconstruction.

In addition to the analyses of the 3D reconstructions, comparisons between the NIR and UV-I datasets and the VIS datasets were performed to identify features specific to the former. These features attracted attention during the first inspection of the multispectral datasets and were used as exemplary findings to illustrate the potential benefits of multispectral 3D imaging. The NIR datasets showed an enhanced visibility of vein patterns. Furthermore, injuries and bruises that presumably were located deeper in the tissue showed noticeable contrast in the NIR datasets. In contrast, signs of subtle bruising on the skin could not be detected in the NIR datasets. The UV-I datasets showed enhanced visibility of foreign substances on the skin. These substances appeared to be antiseptic cleansing agents, adhesives and bodily fluids. Overall, these findings seem consistent with the scientific literature. However, debate about the validity of UV, NIR, and narrowband light sources, especially for diagnosing latent and subtle bruising, appears ongoing [36, 38–40]. To gain a deeper understanding of the validity of different light sources, further analysis is necessary. Such analysis is beyond the scope of the present study. The demonstration of potential benefits of multispectral imaging in this study was based solely on visual comparisons among 3D models of multispectral datasets. These findings were not investigated in detail.

This study demonstrated the possibility of using multispectral photogrammetry for postmortem full-body documentation. Furthermore, it highlighted some of the benefits of 3D UV-I and NIR imaging compared to standard postmortem 3D surface documentation. The imaging setup used in this study was designed based on our experience in our previous projects [13, 15, 20]. In this study, the total number of cameras was reduced to a minimum to introduce an affordable and low-cost approach for 3D multispectral full-body imaging. The setup and the imaging workflow were employed in this study in conjunction with the use of a medical CT scanner. This approach allows the 3D data to be aligned and merged with the corresponding CT data [6, 7, 10, 16, 24]. However, the system is not limited to use in combination with CT scanners. The multicamera rig is movable and can be used to capture photogrammetric datasets in combination with autopsy tables, lifting carts, examination couches and potentially even hospital beds to document postmortem and antemortem cases. The multicamera approach not only facilitates and automates the imaging procedure but also contributes to reproducibility. Furthermore, it allows the implementation of multispectral photogrammetry with similar camera angles and camera positions throughout the entire spectral range. These features enable the user to compare and analyze multispectral data in both 3D and raw form (i.e., 2D photographs). Overall, finding and documenting evidence is key for any forensic investigation. With the help of fast 3D multispectral full-body imaging, the detection of latent evidence can be achieved digitally to reduce the examination time of the body. Furthermore, 3D multispectral full-body imaging allows the storage and preservation of forensically relevant information on the entire body.

The presented setup is a prototype and has not yet been used in routine investigations. Currently, the manual replacement of light sources and lens filters demands manual adjustments and requires the training of personnel to use the system. Furthermore, an improved workflow for multispectral white balancing would help improve the data. In addition to addressing the technical aspects of the imaging setup, future studies should endeavor to include a larger and more diverse set of example cases. Moreover, to further investigate the potential of NIR and UV-I imaging, future projects should include additional methods to analyze the findings.

## Conclusion

In this study, a multicamera setup based on four modified DSLR cameras was designed that can be used in conjunction with a medical CT scanner to semi automatically perform full-body photogrammetry. The use of NIR and UV light sources in combination with appropriate lens filters can allow multispectral photogrammetry to be performed for forensic investigations. The obtained 3D multispectral full-body data can facilitate the detection of latent evidence that is invisible to the naked eye and allow visualization, documentation and analysis of evidence beyond that in the VIS spectrum.

## Key points

1. VirtoScan is a multicamera setup that can be used to perform 3D multispectral full-body imaging.
2. The multispectral setup comprises four modified DSLR cameras, remote shutter controls, UV and NIR light sources and supplemental lens filters.
3. Three-dimensional multispectral full-body imaging is based on close-range photogrammetry and can be automated in combination with a medical CT scanner.
4. Three-dimensional full-body surface documentation should be expanded towards UV and NIR spectra to facilitate the detection, visualization and documentation of latent evidence.
5. Three-dimensional multispectral imaging allows latent evidence to be searched for digitally, making the body available for other examinations.
